# Comparison of Long Non-Coding RNA Expressions in Endometrial Polyp and Endometrial Cancer Cases

**DOI:** 10.3390/diagnostics15212741

**Published:** 2025-10-29

**Authors:** Cagla Bahar Bulbul, Ayla Solmaz Avcikurt, Cagla Kayabasi, Aysegul Dalmizrak, Zafer Erol

**Affiliations:** 1 Department of Obstetrics & Gynecology, Balikesir Ataturk City and Research Hospital, 10100 Balikesir, Türkiye; 2 Department of Medical Biology, Balikesir University, 10145 Balikesir, Türkiyekayabasicagla@gmail.com (C.K.); ayseguldalmizrak@gmail.com (A.D.); 3 Department of Medical Pathology, Balikesir Ataturk City and Research Hospital, 10100 Balikesir, Türkiye

**Keywords:** endometrial cancer, endometrial polyp, long non-coding RNA, UCA1, MALAT-1, XIST

## Abstract

**Aim**: This study compared the expression of four long non-coding RNAs (lncRNAs)—XIST, UCA1, MALAT1, and ANRIL—in endometrial polyps (EP), endometrial cancer (EC), and normal endometrium to assess their diagnostic and prognostic potential. **Materials and Methods**: In this prospective study, 150 women undergoing endometrial biopsy between August 2021 and April 2024 were included (50 EP, 50 EC, 50 controls). RNA was extracted from FFPE tissue, converted to cDNA, and analyzed using SYBR Green-based qRT-PCR with U6 snRNA as reference. Statistical analysis included ANOVA/Kruskal–Wallis, logistic regression, and ROC analysis; *p* < 0.05 and fold change ≥±2 were considered significant. **Results**: The mean age was significantly higher in EC than in EP and controls (*p* < 0.05), with BMI also elevated (*p* = 0.006). UCA1 expression was upregulated in EP compared with controls (*p* = 0.008) but markedly downregulated in EC (*p* < 0.0005). XIST, MALAT1, and ANRIL showed upward trends in EC without independent statistical significance. Logistic regression identified age and UCA1 as the only independent predictors. Diagnostic accuracy was high: EC vs. control AUC = 0.98; EP vs. control AUC = 0.86; EC vs. EP AUC = 0.87. Age predicted malignancy, while high UCA1 was associated with EP and low UCA1 with EC. **Discussion**: Age and UCA1 expression were the strongest discriminators between lesion types. UCA1’s dual, context-dependent role—promoting benign proliferation in EP and decreasing in EC—suggests potential biomarker utility. Other lncRNAs aligned with oncogenic functions but lacked independent predictive value. Combining molecular and clinical parameters could improve risk stratification and early detection, warranting validation in larger cohorts.

## 1. Introduction

The endometrium is the innermost lining of the uterus and plays a central role in the reproductive physiology of women. From menarche to menopause, it undergoes cyclic and dynamic changes under the influence of the two most critical hormones in female health: estrogen and progesterone [1,2]. However, this dynamic nature also makes it susceptible to various pathological conditions. Endometrial polyps (EP) and endometrial cancer (EC) are two of the most frequently encountered conditions, and there is biological and molecular interplay between them [3,4].

EPs are predominantly benign lesions characterized by hyperplasia of glandular and stromal tissues [5]. Their incidence increases with age and has been reported to range between 10 and 24% in the general population [6]. EPs are often asymptomatic. However, especially in reproductive-aged, premenopausal, and postmenopausal women, they may manifest as abnormal uterine bleeding, including menorrhagia, metrorrhagia, or intermenstrual bleeding [7,8]. Although endometrial polyps are generally benign, some may exhibit premalignant changes or coexist with malignant lesions, with the range between 0.8% and 10% [9,10,11]. In addition to histological examination, the use of molecular markers like long non-coding RNAs could enhance early and accurate prediction of malignancy potential [12,13].

EC is a common gynecological malignancy arising from estrogen-stimulated endometrial hyperplasia, often due to unopposed estrogen exposure. As of 2020, it accounted for ~4.5% of all female cancers, with 417,367 new cases globally [14]. Major risk factors are obesity, type 2 diabetes, hypertension, chronic anovulation, and prolonged estrogen exposure [15]. It has a high survival rate when diagnosed in its early stages [16]. However, in stage III–IV disease, survival drops dramatically. Therefore, early diagnosis, risk-stratified treatment, and molecularly targeted therapies are of vital importance.

In recent years, research focusing on molecular signaling pathways and non-coding RNAs has yielded significant advancements [17]. Long non-coding RNAs (lncRNAs), which are over 200 nucleotides in length and regulate gene expression at both transcriptional and post-transcriptional levels, have been shown to play important roles in cancer biology [18]. LncRNAs participate in processes such as cell proliferation, apoptosis, invasion, and metastasis, and have been studied as potential biomarkers or therapeutic targets in various cancer types [18,19]. In particular, changes in lncRNA expression in premalignant lesions (such as atypical polyps or hyperplasia) have been proposed as early biomarkers for malignant transformation [12,18,20]. Therefore, comparative evaluation of lncRNA expression profiles between EP and EC may provide critical insight into early malignancy detection and biomarker development.

XIST is frequently upregulated in endometrial, cervical, and ovarian cancers, where it modulates cell proliferation, migration, and epithelial–mesenchymal transition (EMT) through the regulation of miRNA-mediated signaling pathways [21,22]. UCA1, conversely, has shown context-dependent behavior—acting as an oncogenic driver in ovarian and cervical carcinomas, but with reports of downregulation in endometrial cancer, suggesting a complex role in endometrial tumorigenesis [10,23,24]. MALAT1 is one of the most extensively studied lncRNAs; its overexpression promotes cell cycle progression, metastasis, and angiogenesis across various gynecologic tumors, including endometrial carcinoma, through modulation of Wnt/β-catenin and PI3K/AKT signaling [25,26,27,28]. ANRIL contributes to cell proliferation and survival by modulating the p15/p16-INK4A tumor suppressor pathway and is associated with poor prognosis in several gynecologic cancers [29,30,31].

In this study, the expression levels of four lncRNAs—XIST, UCA1, MALAT1, and ANRIL—that have been previously associated with proliferation, apoptosis, angiogenesis, and metastasis in EP and EC biology were evaluated in comparison with healthy controls (proliferative or secretory endometrium). The main objective was to assess the discriminative potential of these lncRNAs between benign and malignant endometrial lesions, and to evaluate their diagnostic and prognostic utility. Based on the findings, we believe that this study could contribute to the molecular understanding of malignant transformation in EP and support the development of personalized management and follow-up strategies in the future.

## 2. Materials and Methods

### 2.1. Study Design and Sample Selection

This prospective observational study with prospectively collected samples and retrospective laboratory analysis was conducted on patients who presented with abnormal uterine bleeding and underwent endometrial biopsy at the Department of Obstetrics and Gynecology, Balıkesir Atatürk City Hospital, between August 2021 and April 2024. Based on histopathological evaluations, a total of 150 patients were included in the study, comprising 50 cases diagnosed with EP, 50 cases diagnosed with endometrial cancer, and 50 patients with proliferative or secretory endometrium as the control group. Demographic data and peripheral blood levels were obtained from the hospital data system.

We included in this study the women aged 18 years and above, not using systemic steroids or hormonal therapy in the last 3 months, and with the absence of other malignancies. Patients with a history of uterine leiomyoma, adenomyosis, or endometriosis were excluded. Informed consent was obtained from all participants, and the study design was prospectively approved by Balıkesir Atatürk City Hospital Ethics Committee (Decision No: 2024/06/29 and date of approval: 13 June 2024). All tissue samples were obtained from formalin-fixed paraffin-embedded (FFPE) blocks provided by the Department of Pathology of the same hospital. The paraffin blocks were sent to the Department of Medical Biology, Balıkesir University, with ethical approval.

### 2.2. RNA Isolation

Total RNA isolation was performed using the EcoSpin FFPE Total RNA Isolation Kit (Cat. No: E2095, Ecotech Biotechnology, Erzurum, Türkiye), optimized for extracting high-purity RNA from FFPE tissue samples. The advantages of this kit include low RNA degradation rate, high yield, and the ability to preserve all RNA types, including lncRNAs. Five sections were prepared from each FFPE block. All steps were performed according to the manufacturer’s instructions for use. The eluted RNA was stored at −80 °C. Total RNA integrity of all samples was confirmed by agarose gel electrophoresis, and only samples with acceptable RNA quality (absorbance ratio of A260/A280 ≥ 2.0) were included in the subsequent analysis.

### 2.3. cDNA Synthesis

Complementary DNA (cDNA) synthesis was performed using the OneScript Plus cDNA Synthesis Kit (Cat. No: G236, Applied Biological Materials, ABM Good, Richmond, BC, Canada). The procedure was carried out in accordance with the manufacturer’s instructions. Prior to reverse transcription, RNA integrity, concentration, and potential contamination were evaluated spectrophotometrically. Reaction mixes were prepared on ice and incubated at 50–55 °C for 15 min.

### 2.4. Quantitative Real-Time PCR (qRT-PCR) for Expression Analysis

The expression levels of the selected lncRNAs were analyzed using qRT-PCR with SYBR Green BlasTaq™ 2X qPCR MasterMix (Cat.No.: G891, Applied Biological Materials, ABM Good, Richmond, BC, Canada) from cDNA templates. U6 snRNA was used as the reference gene. The primer sequences for the four lncRNAs and U6 in the RT-qPCR have been added as “Supplementary Table S1 (Primer sequences used for RT-qPCR)”. All reactions were conducted in technical triplicates (Table 1).

### 2.5. Statistical Analysis

First, the Shapiro–Wilk test was used to assess normality of data distribution (*p* > 0.05 was considered indicative of normal distribution). Then, Levene’s test was applied to assess the homogeneity of variances (*p* > 0.05 accepted as homogeneous). For data with homogeneous distribution, one-way ANOVA was used; for non-homogeneous distributions, the Kruskal–Wallis H test was applied. If the overall difference was significant, post hoc analysis (pairwise comparisons) tests were performed to determine which groups the difference was between (Tukey HSD test and Mann–Whitney U test with Bonferroni correction). Relative expression levels were calculated using the 2^−ΔΔCt^ method after normalization to U6 snRNA; all reactions were performed in triplicate.

Regression analysis was employed to evaluate correlations among lncRNA expressions. False Discovery Rate correction was used for multiple comparisons. A *p*-value < 0.05, Bonferroni 0.0125 and a fold change ≥±2 were considered statistically significant. Expression levels were normalized using the 2^−ΔΔCt^ method. All statistical analyses were conducted using IBM SPSS Statistics (version 26.0, New Orchard Road Armonk, New York, NY, USA).

## 3. Results

The average age of the groups was as follows: EP group 47.76 ± 9.35 years, endometrial cancer group 59.18 ± 9.2 years, and control group 44.24 ± 5.12 years. One-way ANOVA showed a statistically significant difference among the groups (F(2147) = 14.37; *p* < 0.05; η^2^ = 0.16). Tukey-HSD post hoc analysis revealed significant differences between EC and EP groups (*p* < 0.05), and EC and control groups (*p* < 0.05), but not between EP and control groups (*p* = 0.049). BMI values were: EP = 27.85 ± 3.74 kg/m^2^; EC = 29.12 ± 3.45 kg/m^2^; Control = 27.37 ± 2.48 kg/m^2^. Shapiro–Wilk and Levene tests confirmed assumptions for ANOVA (*p* > 0.05). One-way ANOVA indicated significant differences (F(2147) = 5.31; *p* = 0.006; η^2^ = 0.07). Tukey-HSD post hoc test revealed differences between EC and EP (*p* = 0.018), and EC and control (*p* = 0.002), but not between EP and control (*p* = 0.44). Gravida and parity data did not follow normal distribution (Shapiro–Wilk *p* < 0.05). Kruskal–Wallis H test showed no significant difference (*p* > 0.05). Chi-square test for smoking showed a significantly higher smoking rate in the control group compared to the others (60%, 40%, 36%; *p* = 0.036, χ^2^ = 6.67, Cramér V = 0.21), indicating a small to moderate association (Table 2). All 50 patients in the endometrial cancer group were diagnosed with endometrioid adenocarcinoma. The pathological diagnosis was reported as FIGO Grade 1 in 41 patients, FIGO Grade 2 in 8 patients, and FIGO Grade 3 in 1 patient. Moreover, all endometrial polyps were benign glandular–stromal lesions, measuring 0.5–2.3 cm (mean 1.4 cm) in diameter, and none exhibited atypical or premalignant histological features.

Routine clinical and biochemical parameters are presented in Table 3 for completeness, although they were not included in diagnostic or predictive analyses.

Multivariable logistic regression between EC vs. Control groups is shown in Table 4. When examining the three groups, we aimed to present each lncRNA expression level in pairwise comparisons. UCA1 expression was upregulated in EP compared with controls (*p* = 0.008) but markedly downregulated in EC (*p* < 0.0005). XIST, MALAT1, and ANRIL showed upward trends in EC without independent statistical significance. Supplementary Table S2 is added to display ΔCt, 2^−ΔΔCt^, and adjusted *p*-values for all lncRNAs.

### 3.1. EC vs. Control

**Figure 1:**
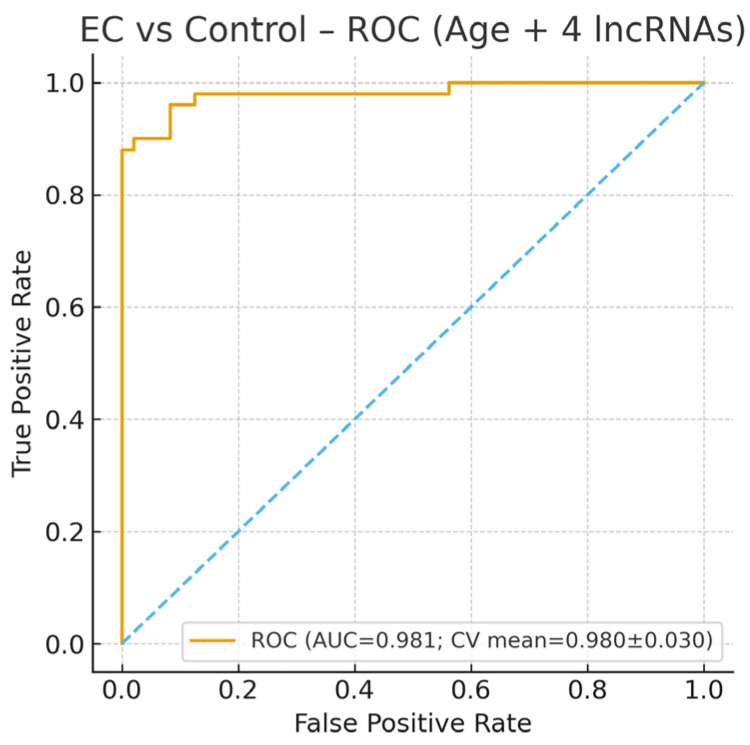
Receiver Operating Characteristic (ROC) curve for the multivariable logistic regression model comparing endometrial cancer (EC) with the control group. The blue dashed line indicates the reference (chance) line corresponding to an area under the curve (AUC) of 0.5, representing random classification performance. The model incorporated age and the expression levels of four long non-coding RNAs (ANRIL, MALAT1, UCA1, and XIST; normalized as 2^−ΔΔCt^). After recalculation from the raw dataset, the model demonstrated excellent diagnostic performance for distinguishing EC from controls, achieving an area under the curve (AUC) = 0.98 (cross-validated AUC = 0.98 ± 0.03). The ROC curve was generated from a five-fold cross-validation to ensure model robustness. These findings confirm that the combination of age and lncRNA expression profiles provides a highly accurate biomarker panel for identifying endometrial cancer cases.

**Figure 2:**
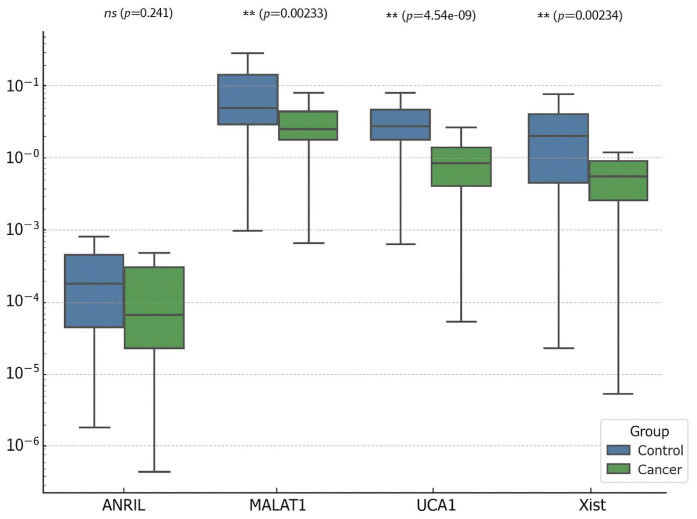
Expression level (2^−ΔCt^) of lncRNAs in endometrial cancer (EC) versus the control group. Boxplots show the relative expression levels (2^−ΔΔCt^) of ANRIL, MALAT1, UCA1, and XIST in endometrial cancer (EC) and control endometrial tissues, normalized to U6 snRNA. The median line represents the central tendency, boxes denote the interquartile range (IQR), and whiskers indicate 1.5× IQR. Statistical analysis was performed using the Mann–Whitney U test with Bonferroni correction for multiple comparisons. Asterisks represent significance levels. UCA1 expression was significantly lower in EC compared with controls, consistent with its potential downregulation in malignant transformation, while MALAT1 and XIST exhibited increased expression trends in EC tissues.

When comparing patients with endometrial cancer and controls, age and UCA1 levels were found to be particularly discriminatory. While the risk of endometrial cancer increases significantly with age, low UCA1 levels may be a protective factor. The model’s AUC value was 0.98, and its explanatory power was high (Nagelkerke R^2^ = 0.56), demonstrating advanced diagnostic power (Figure 1). These findings suggest that molecular markers may play an important role in clinical risk assessment. And Figure 2 also shows the relative expression (2^−ΔΔCt^) of lncRNAs in endometrial cancer (EC) versus the control group.

Conversely, the expressions of ANRIL, MALAT1, and XIST did not show statistically significant associations in the multivariate model, despite trends toward upregulation. These findings suggest that while these lncRNAs may be involved in the pathogenesis of endometrial cancer, UCA1 and age serve as the most robust independent markers in this model.

The model’s explanatory power, with a Nagelkerke R^2^ of 0.56, supports the relevance of these variables in differentiating malignant from non-malignant endometrial tissue. Moreover, all VIF values were below 2, ruling out multicollinearity among predictors.

### 3.2. EP vs. Control

**Figure 3:**
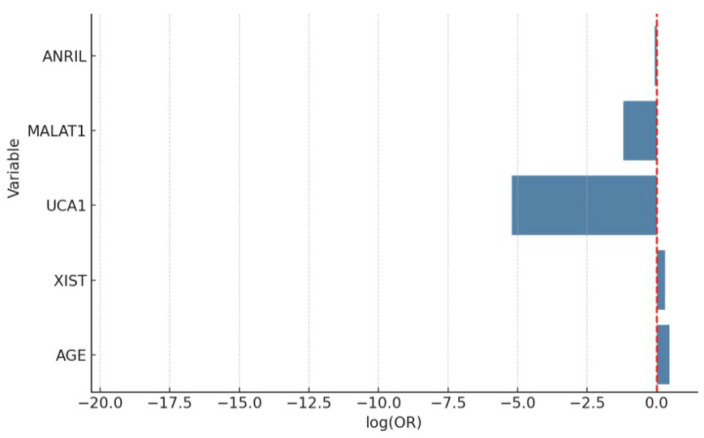
Bar plot of log-transformed odds ratios (ORs) with confidence intervals for variables predicting endometrial cancer. The red dashed line represents the reference line at log(OR) = 0, indicating no effect (neutral association) between the variable and the outcome. According to the multivariate logistic regression analysis, age was identified as a strong independent risk factor for endometrial cancer (OR = 1.58, 95% CI: 1.22–2.04, *p* = 0.0003). Interestingly, UCA1 expression showed a significant inverse association with EC, suggesting its potential role as a negative biomarker (OR = 0.0055, 95% CI: 0.0002–0.26, *p* = 0.011). Other lncRNAs did not demonstrate independent predictive value when adjusted for age.

**Figure 4:**
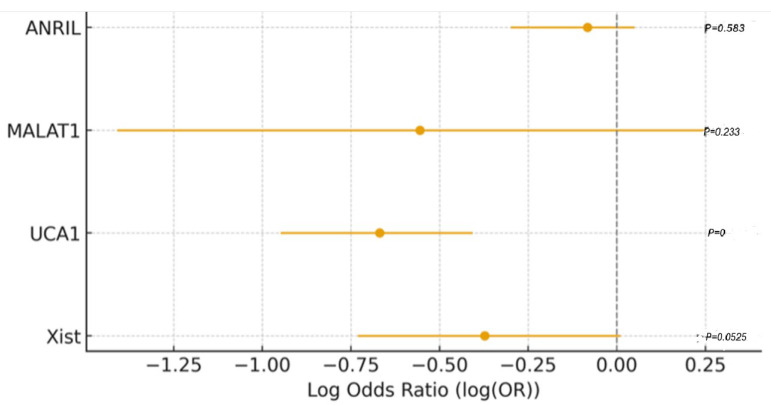
Log odds ratios (95% CI) of lncRNA expression in endometrial polyp (EP) versus control group. Log (OR) plot shows multivariate logistic regression analysis results including ANRIL, MALAT1, UCA1, and XIST. The X-axis represents the log odds ratio (log (OR)) with 95% confidence intervals; the vertical dashed line indicates the neutral value (log (OR) = 0). Positive log (OR) values indicate higher expression associated with EP, whereas negative values suggest downregulation in EP relative to controls. Among the analyzed lncRNAs, UCA1 demonstrated a significantly negative log (OR), implying that lower UCA1 expression is associated with the presence of endometrial polyps, while XIST showed a borderline trend toward downregulation. Other lncRNAs (ANRIL and MALAT1) did not show statistically significant associations after correction. Receiver Operating Characteristic (ROC) curve for the logistic regression model comparing endometrial polyp (EP) vs. control group. The logistic regression model for distinguishing EP from control yielded an AUC of 0.86, sensitivity of 80.0%, specificity of 75.6%, accuracy of 77.9%, precision of 78.3%, and F1-score of 0.79. The optimal threshold (Youden Index) was 0.653 with a maximum index of 0.640.

**Figure 5:**
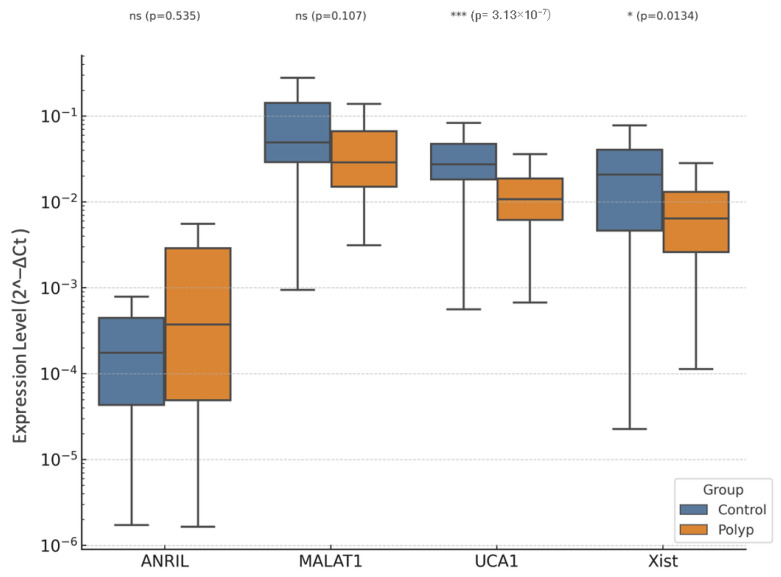
Boxplots of expression levels of long non-coding RNAs (ANRIL, MALAT1, UCA1, and XIST) in endometrial polyp (EP) and control groups. Boxplots represent the relative expression (2^−ΔCt^) of ANRIL, MALAT1, UCA1, and XIST, normalized to U6 snRNA. The median line represents the central tendency, boxes denote interquartile ranges (IQR), and whiskers indicate 1.5× IQR. Statistical comparisons between the EP and control groups were performed using the Mann–Whitney U test with Bonferroni correction. Asterisks indicate significance levels (* *p* < 0.05, *** *p* < 0.001). UCA1 and XIST were significantly up-regulated in EP tissues compared with controls, suggesting their possible role in cell proliferation and benign endometrial growth, whereas ANRIL and MALAT1 showed mild, non-significant increases. Boxplot of lncRNA expression levels (ANRIL, MALAT1, UCA1, XIST) in endometrial polyp (EP) versus control group.

In a comparison between patients with EP and controls, between lncRNAs, levels showed significant differences (Figure 3, Figure 4 and Figure 5).

**Figure 6:**
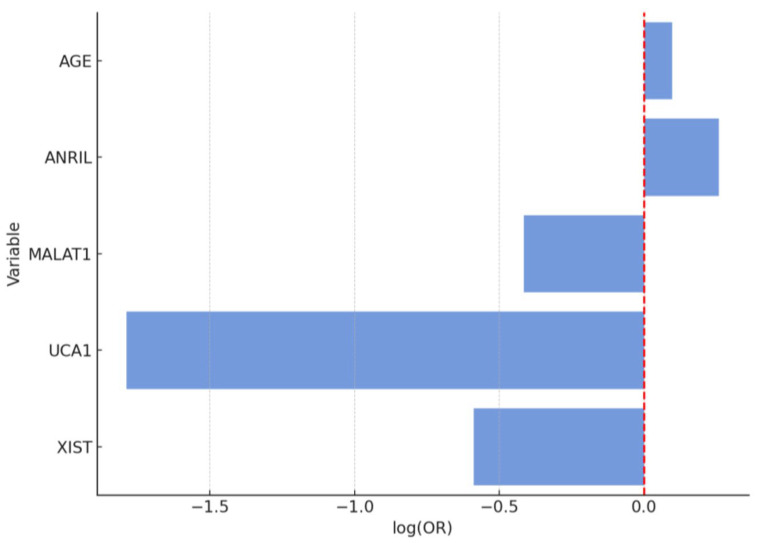
Bar plot of log-transformed odds ratios (ORs) for variables predicting endometrial polyps. The red dashed line represents the reference line at log(OR) = 0, indicating no effect (neutral association) between the variable and the outcome.

While elevated XIST levels were associated with the presence of EP, demonstrating a protective effect and XIST showing a positive association with EP (OR > 1), low UCA1 levels may have a protective effect against polyps (OR < 1) (Figure 6). Interestingly, age was also found to be a significant predictor, with older patients showing increased odds of having EP, in line with known epidemiological trends that link EP with perimenopausal hormonal dynamics.

### 3.3. EC vs. EP

**Figure 7:**
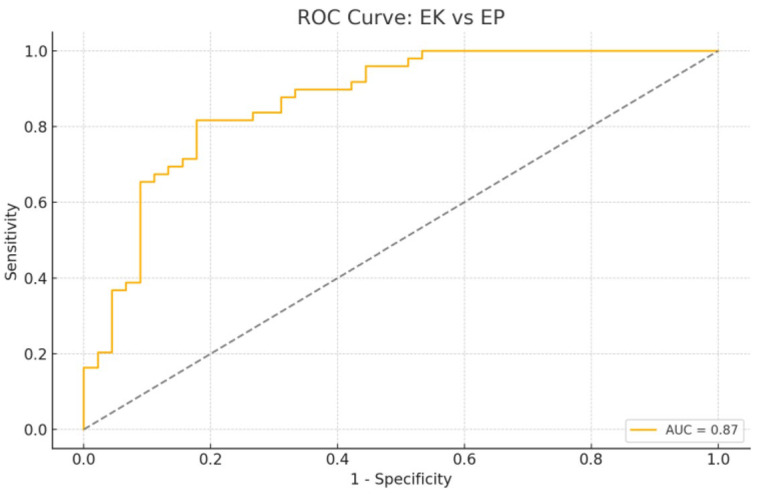
Receiver Operating Characteristic (ROC) curve for the logistic regression model comparing endometrial cancer (EC) vs. endometrial polyp (EP) group. The dashed diagonal line represents the reference (chance) line (AUC = 0.5), indicating no discriminative ability (random classifier). The logistic regression model for distinguishing EC from EP yielded an AUC of 0.87, sensitivity of 81.6%, specificity of 73.3%, accuracy of 77.7%, precision of 76.9%, and F1-score of 0.79. The optimal threshold (Youden Index) was 0.577 with a maximum index of 0.639.

**Figure 8:**
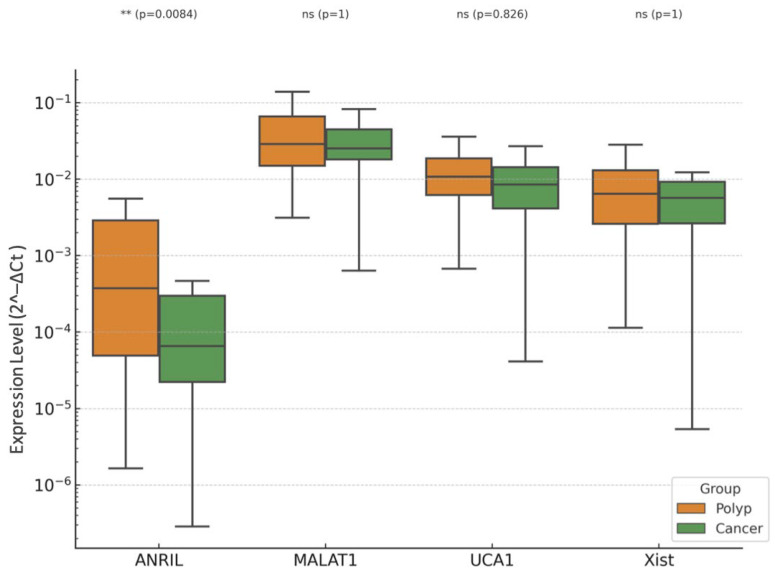
Boxplots of expression levels of long non-coding RNAs (ANRIL, MALAT1, UCA1, and XIST) in endometrial cancer (EC) and endometrial polyp (EP) groups. Boxplots display the relative expression (2^−ΔCt^) of ANRIL, MALAT1, UCA1, and XIST, normalized to U6 snRNA. The median line indicates the central tendency, boxes represent the interquartile range (IQR), and whiskers denote 1.5× IQR. Statistical analysis between EC and EP groups was performed using the Mann–Whitney U test with Bonferroni correction. Asterisks indicate statistical significance (** *p* < 0.01). Although most comparisons did not reach statistical significance, MALAT1 and XIST tended to show higher expression in EC, whereas UCA1 expression was lower, suggesting their differential involvement in benign versus malignant endometrial pathogenesis. Boxplot of lncRNA expression levels (ANRIL, MALAT1, UCA1, XIST) in endometrial cancer (EC) versus endometrial polyp (EP) group.

In the comparative analysis of EC and EP groups, age emerged as the most powerful and statistically significant variable associated with malignancy (Figure 7 and Figure 8). This aligns with well-established epidemiological data showing that endometrial cancer risk increases with advancing age, especially in postmenopausal women due to prolonged unopposed estrogen exposure and declining progesterone levels.

**Figure 9:**
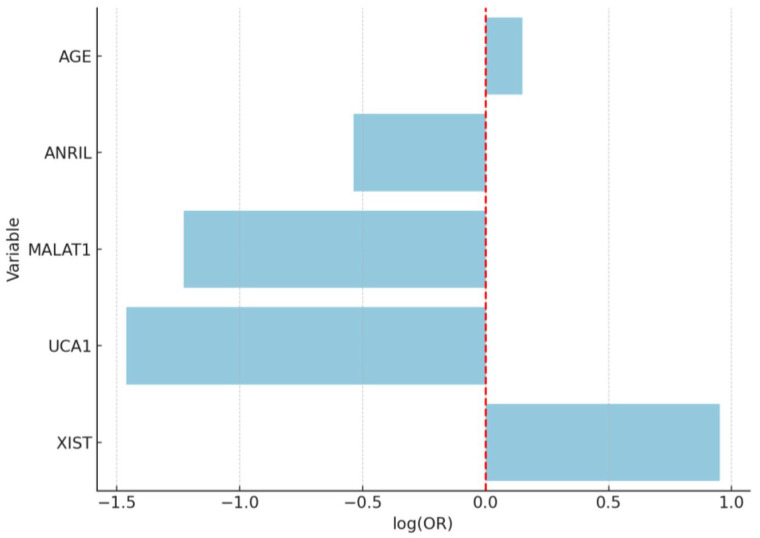
Bar plot of log-transformed odds ratios (ORs) for variables predicting endometrial cancer (EC) versus EP. The red dashed line represents the reference line at log(OR) = 0, indicating no effect (neutral association) between the variable and the outcome.

Despite noticeable trends in lncRNA expression, including upregulation of XIST and MALAT1 in the EC group, none of the lncRNAs evaluated (ANRIL, MALAT1, UCA1, XIST) reached statistical significance in the multivariate logistic regression model (Figure 9). This may be due to overlapping expression profiles between polypoid lesions and malignant tissues, especially in preinvasive or early-stage disease, where molecular distinctions are subtler. It is also possible that the carcinogenic transformation involves additional regulatory layers beyond the lncRNAs included in this panel, such as microRNAs, chromatin remodeling, or epigenetic silencing.

## 4. Discussion

While cellular proliferation is essential for tissue regeneration and developmental processes in organisms, uncontrolled and dysregulated proliferation underlies the pathophysiological mechanisms of many diseases, including cancer. In cancer biology, lncRNAs have been shown to play significant roles in regulating processes such as cellular proliferation, apoptosis, angiogenesis, and metastasis [12,18,19]. The present study investigated the expression profiles of four lncRNAs-XIST, MALAT1, UCA1, and ANRIL-in EP, EC and normal endometrium, with the aim of elucidating their potential diagnostic and prognostic value in differentiating benign from malignant lesions. Our findings highlight that age and UCA1 expression are the most powerful independent predictors of lesion type, while other lncRNAs demonstrated directional changes consistent with previous literature but did not reach statistical significance in multivariate analyses. Age-related epigenetic modulation may partly explain the differential lncRNA expression patterns observed between benign and malignant endometrial lesions. Aging has been associated with altered DNA methylation and estrogen-responsive chromatin remodeling, which could indirectly affect lncRNA transcription. This mechanism has been discussed as a possible link between menopausal hormonal status and transcriptomic reprogramming in endometrial tissue.

Approximately 10–15% of postmenopausal bleeding cases are attributed to EP [32]. While most EPs exhibit a benign course, they are included among lesions with a 1–3% risk of malignant transformation. Therefore, their molecular characterization is crucial in predicting this risk [11,33]. LncRNA expression in EP may facilitate molecular distinction between benign and premalignant polyps and serve as a basis for non-invasive biomarker development [20,34]. The studies have highlighted important roles for lncRNAs in the pathogenesis of EC [35,36,37].

XIST plays a role in X chromosome inactivation and epigenetic regulation. Abnormal expression of XIST in EP may disrupt stromal cell proliferation or the balance of apoptosis. Additionally, XIST is thought to be associated with hormonal microenvironmental changes in polyps [21]. Our study found a trend toward higher XIST expression in EC, aligning with these reports, although statistical significance was not reached.

UCA1, which is involved in the activation of proliferative signaling pathways (PI3K/AKT, Wnt/β-catenin), is proposed to accelerate the cell cycle in the stroma of polyps. UCA1 is implicated in cell cycle progression, angiogenesis, and adaptation to hypoxia. Its upregulation in EP may promote benign proliferation through PI3K/AKT/mTOR signaling and VEGF-mediated angiogenesis. Conversely, its downregulation in EC may reflect stage-specific shifts in tumor biology or activation of alternative growth pathways.

MALAT1, which affects alternative splicing, may enhance the proliferative capacity of both the epithelial and stromal compartments of polyps. Due to its supportive effects on cell migration and invasion, increased MALAT1 expression in polyps is hypothesized to contribute to malignant potential [38,39]. MALAT1 has been shown to enhance proliferation, migration, and invasion in EC cells. Our data showed non-significant upward trends in EC, which may become significant in larger cohorts.

ANRIL, which epigenetically represses the CDKN2A/B locus, may lead to loss of cellular control in the stromal compartment of polyps by downregulating tumor suppressor genes such as p15 and p16. This mechanism may underlie the molecular basis of premalignant changes in polyps [31]. ANRIL induces proliferation by epigenetically silencing the transcription of tumor suppressor genes (p15, p16, ARF) located in the CDKN2A/B locus. While our study observed modest elevation in EC, it did not achieve significance, possibly due to small effect size or sample heterogeneity.

Our data demonstrate that age was significantly higher in the EC group than in both EP and control groups, reaffirming its established role as a strong risk factor for endometrial malignancy [15]. Importantly, UCA1 showed a distinct pattern: it was significantly upregulated in EP compared with controls but significantly downregulated in EC compared with both EP and controls. This suggests a potential context-dependent, dual role, supporting proliferation in benign hyperplasia while being suppressed or displaced by alternative oncogenic drivers in cancer. Other lncRNAs showed directional changes consistent with tumor-promoting activity but did not independently predict disease category when adjusted for age and UCA1.

Although the predictive model was primarily driven by age, the subtle differential expression trends observed in lncRNAs, particularly XIST, warrant further investigation. It is plausible that lncRNAs may have additive or synergistic effects in combination with hormonal and genetic pathways in the malignant transformation of endometrial tissue.

In the EP vs. control comparison, elevated UCA1 emerged as an independent predictor, with an AUC of 0.86. This suggests its involvement in benign endometrial proliferation, potentially via PI3K/AKT and Wnt/β-catenin activation, enhancement of anti-apoptotic signaling, and promotion of stromal cell survival [24,40]. These pathways are discussed as potential mechanisms based on previous literature; no protein-level validation was performed in this study.

XIST expression showed an upward trend in EP, though without statistical significance. This is consistent with its proposed role in modulating stromal–epithelial balance through epigenetic regulation and possible responsiveness to perimenopausal hormonal changes [21]. The combination of older age and elevated UCA1 in EP patients aligns with epidemiological data linking perimenopause to polyp development via unopposed estrogen exposure.

In EC vs. control, age remained the strongest predictor (OR = 1.58, *p* < 0.05). UCA1 expression was significantly lower in EC, acting as a negative predictor of malignancy. This inverse association may indicate that in malignant transformation, other oncogenic pathways, possibly involving MALAT1-mediated epithelial–mesenchymal transition (EMT) or XIST-mediated miRNA sponging, predominate, while UCA1’s proliferative role becomes redundant or even growth-restrictive. While ANRIL, MALAT1, and XIST were upregulated in EC compared with controls, none reached statistical significance in the multivariate setting, likely due to overlapping expression patterns in early malignant and benign hyperplastic lesions.

Direct comparison of EC and EP revealed that age again dominated the model’s predictive capacity (AUC = 0.87), with lncRNAs failing to independently discriminate between groups. This suggests that molecular alterations in early-stage EC may be subtle and partially shared with polypoid lesions, especially those with atypical hyperplasia. It also underscores the diagnostic challenge of distinguishing high-risk polyps from early malignancies based solely on limited lncRNA panels.

These results suggest that lncRNAs may not act in isolation, but as part of a broader regulatory network. The findings also underscore the diagnostic value of UCA1, particularly in distinguishing benign polyps from malignancy. The dual behavior of UCA1 across comparisons supports its context-specific role, which has also been suggested in other gynecologic and non-gynecologic cancers. Meanwhile, MALAT1 and XIST, previously associated with cellular proliferation, migration, and EMT, may contribute to disease progression but require validation in larger cohorts.

Overall, this analysis supports the integration of age as a key risk factor and highlights the potential-but not yet definitive-diagnostic utility of select lncRNAs in distinguishing EC from benign polypoid lesions. Larger studies with more comprehensive transcriptomic profiling may help clarify the biological significance of these molecular markers in endometrial pathology.

**Figure 10:**
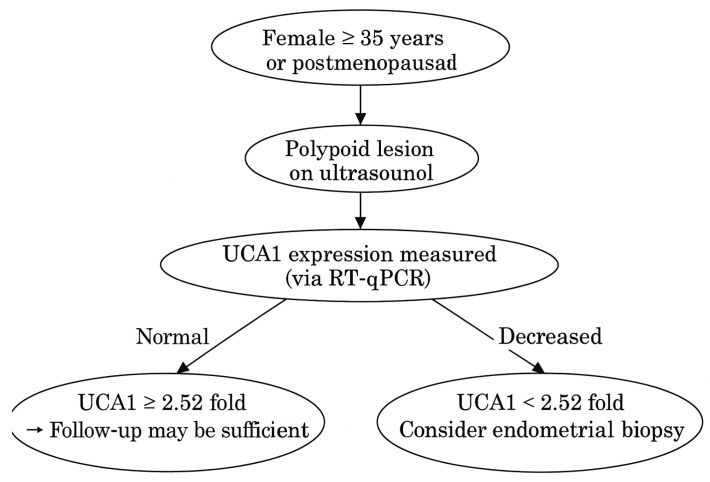
Clinical decision-making algorithm based on UCA1 expression and patient age. This diagnostic flowchart provides clinical guidance for evaluating endometrial lesions based on patient age and UCA1 expression levels. Our findings demonstrated that UCA1 expression is a potential diagnostic indicator for distinguishing between endometrial polyps and cancer, with a cut-off value determined at 2.52-fold. In women aged 35 years or older, or in the postmenopausal period, the detection of a polypoid lesion on ultrasound should prompt further molecular evaluation. If UCA1 expression is decreased (<2.52 fold), the likelihood of malignancy increases, and endometrial biopsy should be considered. This algorithm offers a molecular-based framework to enhance diagnostic decision-making in gynecologic practice.

These findings suggest that lncRNAs may serve as molecular regulators of cell proliferation in EC and highlight their potential importance as diagnostic and prognostic biomarkers. The combination of age and UCA1 expression demonstrated high discriminative ability between benign and malignant lesions, and between polyps and normal endometrium. If validated in liquid biopsies (e.g., plasma, uterine lavage), this biomarker pairing could aid in non-invasive screening and triage of patients with abnormal uterine bleeding. The bidirectional behavior of UCA1 suggests its potential utility as a progression marker in longitudinal monitoring of polyps. We also present the diagnostic algorithm based on age and UCA1 expression in Figure 10. The optimal cut-off value for UCA1 expression to discriminate EC from EP and control tissues was determined by ROC curve analysis using the Youden Index. The cut-off point was identified at a 2.52-fold change (2^−ΔΔCt^), yielding a sensitivity of 82.4%, specificity of 78.6%, and an AUC of 0.98 (95% CI: 0.81–0.92, *p* < 0.001). This supports its potential utility as a molecular marker in differentiating benign from malignant endometrial lesions.

The strengths of our study were the use of comparative sample groups (polyp, cancer, normal endometrium), the evaluation of four distinct lncRNAs, the application of standardized RNA isolation and analysis methods. However, several limitations should be acknowledged, such as potential degradation due to the use of FFPE material for RNA isolation, inability to perform detailed correlations with clinical-parametric data (e.g., stage, grade), and lack of validation at the protein level. Potential model overfitting was addressed through bootstrap validation and cross-validation procedures. Age remained a significant predictor, likely reflecting both biological and statistical confounding related to menopausal hormonal changes. Tumor cellularity, molecular subtypes, and staging data were not uniformly available and are acknowledged as study limitations. Future studies, including independent validation cohorts and multi-center datasets, are warranted to confirm these findings and reduce potential overfitting bias. Detailed information on tumor cellularity, FIGO stage, and molecular subtypes was not available for all archival FFPE specimens, as these data were not uniformly included in the original pathology reports. Only the histological grade could be retrieved and was, therefore, reported. Additionally, pathway enrichment and bioinformatics analyses could further illuminate the regulatory roles of these lncRNAs and their downstream targets.

## 5. Conclusions

In this study, the expression levels of the lncRNAs XIST, MALAT1, UCA1, and ANRIL were compared in samples obtained from EP, EC, and normal endometrial tissues. Although the roles of these four lncRNAs in various cancer types have been demonstrated in the literature, data regarding their differential expression between EP and EC remain limited. In conclusion, this study identifies UCA1 and age as the most informative markers for differentiating benign and malignant endometrial lesions. The dual role of UCA1-upregulated in EP, downregulated in EC, suggests a context-dependent function in endometrial biology. XIST, MALAT1, and ANRIL showed non-significant upward trends, which may reflect secondary or context-dependent changes rather than independent oncogenic effects; they lacked independent predictive value in this cohort. These results provide a foundation for integrating lncRNA profiling into diagnostic algorithms, with UCA1 emerging as a promising candidate for future translational applications.

Based on these findings, lncRNA profiling may provide molecular-level information in addition to traditional histopathological diagnosis, enhance diagnostic accuracy, aid in identifying high-risk polyps, and offer new possibilities for the early diagnosis, prognostic evaluation, and targeted therapy development for EC. Validation of these findings through larger patient series and functional studies in the future is essential to pave the way for the clinical application of lncRNAs.

## Figures and Tables

**Table 1:** Thermal Cycling Conditions.

Step	Temperature	Time	Cycles
Enzyme activation	95 °C	3 min	1
Denaturation	95 °C	15 s	40
Annealing/Extension	60 °C	1 min	40

**Table 2:** Demographic characteristics.

Variable	EP	EC	Control	*p*-Value
Age (years) *	47.76 ± 9.35	59.18 ± 9.2	44.24 ± 5.12	<0.001
BMI (kg/m^2^)	27.85 ± 3.74	29.12 ± 3.45	27.37 ± 2.48	0.0127
Gravida (n)	1.68 ± 0.84	2.26 ± 1.27	1.78 ± 0.93	0.0117
Parity (n)	1.42 ± 0.84	1.86 ± 1.13	1.52 ± 0.84	0.0626
Smoking (%)	36%	40%	60%	0.0361

* ANOVA (*EP: Endometrial polyp; EC: Endometrial cancer; BMI: Body Mass Index*).

**Table 3:** Clinical and biochemical characteristics.

Variable	EP	EC	Control	*p*-Value
Ferritin (ng/mL)	58.73 ± 82.97	37.27 ± 40.72	51.13 ± 56.97	0.9152
Glucose (mg/dL)	81.53 ± 6.75	78.26 ± 10.84	87.12 ± 15.4	0.0123
Haemoglobin (g/dL)	12.11 ± 1.17	11.33 ± 1.23	11.76 ± 1.28	0.007
WBC (K/μL)	7.73 ± 1.83	7.59 ± 2.39	7.39 ± 1.92	0.6837
Lymphocyte (K/μL) *	2.12 ± 0.46	1.99 ± 0.58	1.89 ± 0.42	0.0674
Neutrophil (K/μL)	6.63 ± 1.81	7.31 ± 2.18	7.16 ± 1.77	0.1615
Platelet (×10^3^)	225.62 ± 76.74	231.34 ± 57.37	202.48 ± 60.3	0.0139
Total Cholesterol (mg/dL)	177.67 ± 34.46	201.05 ± 43.21	212.6 ± 71.59	0.060
HDL (mg/dL)	63.17 ± 12.82	56.29 ± 9.37	55.74 ± 15.5	0.1046
LDL (mg/dL)	93.77 ± 35.48	112.16 ± 34.06	105.71 ± 41.32	0.0874
Triglyceride (mg/dL)	137.43 ± 63.76	168.11 ± 92.5	207.37 ± 96.1	0.0063
Free T3 (pg/mL) *	3.3 ± 0.8	3.14 ± 0.72	3.17 ± 0.69	0.7322
Free T4 (ng/dL)	1.35 ± 0.35	1.19 ± 0.25	1.31 ± 0.3	0.2432
TSH (uIU/mL)	3.42 ± 2.17	3.58 ± 2.26	3.31 ± 2.74	0.7425
Creatinin (mg/dL)	1.32 ± 0.49	1.24 ± 0.5	1.09 ± 0.36	0.0638
Urea (mg/dL)	31.08 ± 13.2	26.8 ± 12.6	25.66 ± 10.28	0.0851
Vitamin D	15.83 ± 25.45	15.27 ± 29.06	19.50 ± 27.46	0.8112

* ANOVA (*WBC: White blood cell; HDL: High density lipoprotein; LDL: Low density lipoprotein; TSH: Thyroid stimulating hormone*).

**Table 4:** Multivariable logistic regression for EC vs. Control.

Variable	Coefficient (Log-Odds)	Std. Error	Wald *p*-Value	OR	95% CI (Low)	95% CI (High)
Intercept	−0.0834	0.1711	0.620	—	—	—
UCA1	1.3945	0.3257	<0.001	4.03	2.13	7.64
XIST	0.8721	0.3493	0.012	2.39	1.21	4.74
MALAT1	0.6517	0.2912	0.025	1.92	1.09	3.38
ANRIL	0.3178	0.2845	0.264	1.37	0.78	2.41
Age	1.0124	0.3025	0.001	2.75	1.52	4.97

Multivariable logistic regression (dependent variable: EC = 1, Control = 0). Predictors standardized; expression normalized as 2^−ΔΔCt^. Predictors: Age + 4 lncRNAs (UCA1, XIST, MALAT1, ANRIL). Data: 2^−ΔΔCt^ expression values standardized (Z-score). AUC: 0.98 (5-fold CV mean 0.98 ± 0.03).

## Data Availability

Dataset available on request from the authors.

## Supplementary Materials

The following supporting information can be downloaded at: https://www.mdpi.com/article/10.3390/diagnostics15212741/s1. Supplementary Table S1: Primer sequences used for RT-qPCR. Supplementary Table S2: Group-wise Expression (2^−ΔCt^) and Statistical Comparisons.
