# Supplementary material for: Comparison of Long Non-Coding RNA Expressions in Endometrial Polyp and Endometrial Cancer Cases
Source: Diagnostics (Basel). 2025 Oct 29;15(21):2741. doi: 10.3390/diagnostics15212741 (PMC12607446; doi:10.3390/diagnostics15212741)
Supplement: Supplementary file 1 [file diagnostics-15-02741-s001.zip › diagnostics-3922044-supplementary.pdf]

Serial Number	Oligo Name	Base Sequence 5′-3′	Purification type	Synthesis Scale (nmol)	Number of Bases	TM (°C)	GC (%)	MW (g/mol)	nmol	A260	OD	μl for 100 μM	Amplicon (bp)
20240826/2-158	UCA1-F	CACTCTTTGCCAGCCTCAGCTT	Desalting	50	22	62	55	6597	45	45	11	448	122
20240826/2-159	UCA1-R	AGGTGTGAGTGGCGGTCTGAAT	Desalting	50	22	62	55	6886	54	56	14	539	122
20240826/2-160	Xist-F	GTAGGTGTGCTGATAACCAAGGC	Desalting	50	23	62	52	7129	52	56	14	522	118
20240826/2-161	Xist-R	GGGAAAGGAAGATTGAGGGTGG	Desalting	50	22	62	55	6994	50	53	13	496	118
20240826/2-162	U6-F	CTCGCTTCGGCAGCACAT	Desalting	50	18	58	61	5436	45	37	9	453	130
20240826/2-163	U6-R	TTTGCGTGTCATCCTTGCG	Desalting	50	19	57	53	5777	47	41	10	466	130
20240826/2-164	Malat1-F	GATCTAGCACAGACCCTTCAC	Desalting	50	21	60	52	6335	50	48	12	497	125
20240826/2-165	Malat1-R	CGACACCATCGTTACCTTGA	Desalting	50	20	57	50	6037	61	56	14	610	125
20240826/2-166	ANRIL-F	ACGGAGTCAACCGTTTCGGGAG	Desalting	50	22	64	59	6800	50	52	13	500	85
20240826/2-167	ANRIL-R	GGTCGGGTGAGAGTGGCAGG	Desalting	50	20	66	70	6319	52	49	12	516	85

Supplementary Table S1. Primer sequences used for RT-qPCR. TM: Melting Temperature; GC: Guanine–Cytosine Content; MW: Molecular Weight; OD: Optical Density

Gene	Comparison	Mean (Group 1) ± SD	Mean (Group 2) ± SD	U	p-value	Bonferroni-adj p
UCA1	EC vs Control	1.11 ± 0.41	0.65 ± 0.28	328.0	<0.0001	<0.0005
	EP vs Control	0.94 ± 0.33	0.65 ± 0.28	462.0	0.008	0.032
	EC vs EP	1.11 ± 0.41	0.94 ± 0.33	1012.0	0.139	0.555
XIST	EC vs Control	1.06 ± 0.35	0.72 ± 0.25	410.0	0.0006	0.0022
	EP vs Control	0.92 ± 0.31	0.72 ± 0.25	496.0	0.0036	0.0144
MALAT1	EC vs Control	1.09 ± 0.40	0.71 ± 0.28	398.0	0.0004	0.0018
	EP vs Control	0.83 ± 0.35	0.71 ± 0.28	660.0	0.034	0.136
ANRIL	EC vs Control	0.92 ± 0.39	0.74 ± 0.30	812.0	0.088	0.352

Supplementary Table S2: Group-wise Expression ($2^{-\Delta Ct}$) and Statistical Comparisons. All p-values two-sided, Mann–Whitney U; Bonferroni correction for four genes ($\alpha = 0.0125$).
